# Suicide Attempts in Malaysia from the Year 1969 to 2011

**DOI:** 10.1155/2014/718367

**Published:** 2014-02-03

**Authors:** Aishvarya Sinniah, T. Maniam, Tian Po Oei, Ponnusamy Subramaniam

**Affiliations:** ^1^Department of Psychiatry, Universiti Kebangsaan Malaysia Medical Centre, 56000 Cheras, Malaysia; ^2^School of Psychology, University of Queensland, Brisbane, QLD 4072, Australia; ^3^Health Psychology Programme, Faculty of Health Sciences, Universiti Kebangsaan Malaysia, 50300 Kuala Lumpur, Malaysia

## Abstract

The aim of this paper is to review the literature on suicide attempts in Malaysia. PsycINFO, PubMed, and Medline databases from 1845 to 2012 and detailed manual search of local official reports from the Ministry of Health and the Malaysian Psychiatric Association and unpublished dissertations from 3 local universities providing postgraduate psychiatric training were included in the current review. A total of 38 studies on suicide attempts in Malaysia were found and reviewed. Twenty-seven (76%) of the studies on suicide attempts were descriptive studies looking at sociodemographic data, psychiatric illnesses, and methods and reasons for suicide attempts. No study has been conducted on treatment and interventions for suicide attempts and the impact of culture was rarely considered. The review showed that in order for researchers, clinicians, and public health policy makers to obtain a better understanding of suicide attempts in Malaysia, more systematic and empirically stringent methodologies and research frameworks need to be used.

## 1. Introduction

Almost one million people die from suicide annually, and the average annual suicide rate is 16 per 100,000 globally [1]. By the year 2020, the WHO estimates that approximately 1.53 million people or nearly 3% of all world deaths would be due to suicide, and 10–20 times more people would attempt suicide worldwide. This represents on average one death every 20 seconds and one attempt every 1-2 seconds [2].

In Malaysia, report on government hospital admissions for attempted suicides and deaths as a consequence of this showed constant rise from the year 1999 to 2007. Patients' ages range mostly from 12 to 24 and female numbers are more than males.

Suicidal behavior is a growing cause for concern in Malaysia since suicide rates have increased by 60% over the past 45 years [3]. Malaysia also has a moderately high suicide rate which is approximately 12 per 100,000, though this figure is an estimate that remains disputed as Maniam and Chan [4] have shown. The suicidal rate is comparable to that of countries such as Singapore with 10 per 100,000 [5]. It is also a growing global public health concern since self-inflicted injuries represent 1.4% of the global burden of disease worldwide in 2002 and are expected to increase to 2.4% by 2020 [1]. In Malaysia, 30,000 family members or friends were estimated to be negatively affected directly or indirectly by suicidal acts every year [6].

With suicidal behavior on the rise in Malaysia, empirical research has an important role to play in shedding more light on this problem and its possible solutions. To date, however, very little systematic review of research on this area has been undertaken. Attempting to address this gap, our paper summarizes the results of a systematic literature review of suicide attempt in Malaysia and identifies future directions for research, public health policy, and clinical intervention.

## 2. Method

Studies on suicide attempt in Malaysia were identified after a comprehensive search of the biggest electronic databases: PsycINFO (1845-present), Medline (1950-present), and PubMed (1951-present). Manual search of local official reports from Ministry of Health and Malaysian Psychiatric Association was also undertaken. Local journals in Malaysia such as the Journal of Medicine and Health Sciences, Malaysian Journal of Psychiatry, and Malaysian Journal of Pathology were also reviewed. Finally, unpublished theses and abstracts presented in conferences were obtained from libraries of 3 major universities in Malaysia with medical schools.

**Figure 1:**
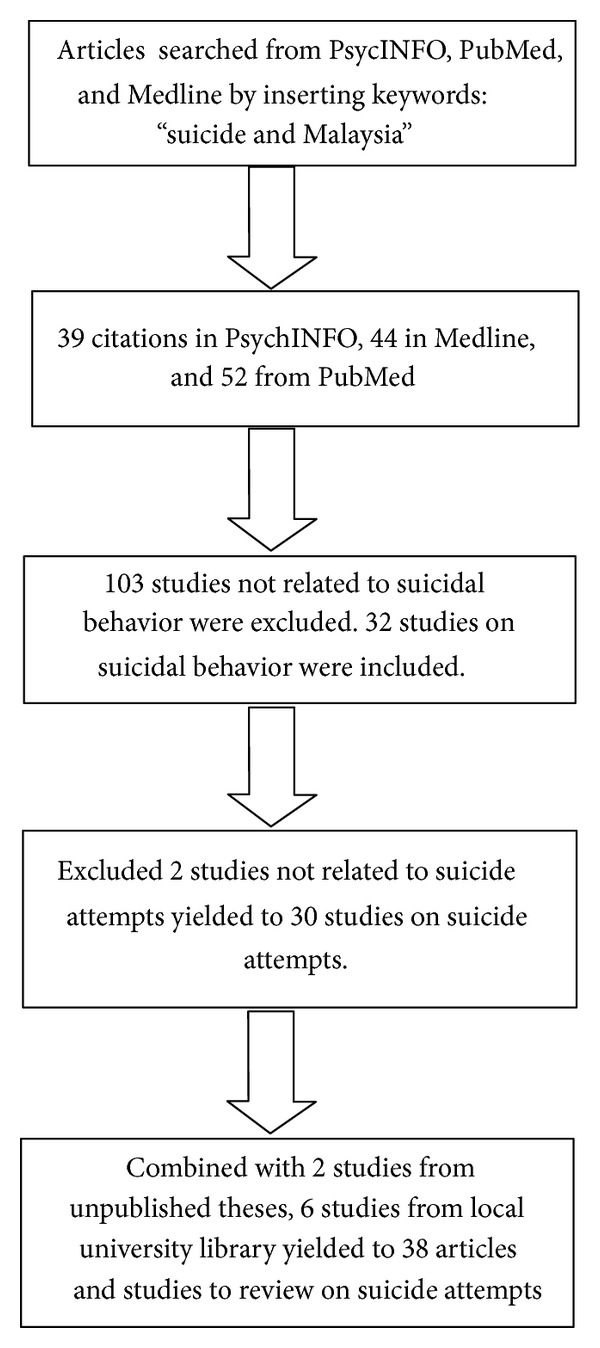
Flow chart of the reviewing process.

Key words used in the search were “suicide and Malaysia.” The use of generic key words was intentional in order to capture as many published papers as possible. These key words were selected by looking at the frequency used in most of the papers collected early in the process of review and they yielded 39 citations in PsycINFO, 44 in Medline, and 52 in PubMed. One hundred and three studies were excluded leaving 32 articles related to suicidal behavior. However, 2 articles on suicidal behavior were excluded since they were not studies on suicide attempt.  Figure 1 presents the process flow that was undertaken for our literature search.

Searches were refined by identifying studies published in English which included descriptive, cross sectional, and experimental studies and reviews. Twenty-nine published journal articles on suicide attempts were found to be suitable for the review. An additional 6 studies from local university libraries and 2 studies from unpublished theses were included. The comprehensive literature search yielded 38 studies on suicide attempts in Malaysia deemed acceptable to be included in the review (Figure 1).

Table 1 presents the following important features of relevant studies: (a) study number and reference, (b) methodology used, (c) number of participants, (d) gender, (e) age, (f) marital status, (g) representative of the ethnicity, (h) education level, (i) employment status, (j) psychiatric diagnosis given, (k) method used for suicidal behavior, and finally (l) reasons for suicidal behavior.

It was important that information gathered from the studies during the review could provide some direction for future research. An asterisk (∗) was used to identify studies that utilized a recognized psychological instrument to measure suicide attempt. This was of particular interest to authors as a way of identifying suicidal behavior scales that have already been used and/or validated for use in the Malaysian context. Study “methodology” (Table 1, column 2) was included to show the range of methodological approaches used by previous researches. Target groups were identified to give a sense of the sample covered by these studies. Sample size is also noted since larger sample sizes give more reliable results than smaller sample sizes (*n* < 30). Since Malaysia is a multiethnic society, ethnicity of study participants was also included. Information on sex and age distribution would also guide recommendations on intervention programs especially in schools. Marital status, education level, and employment status information were reported to identify trends that may possibly indicate whether these can indeed be protective factors behind suicidal behavior as reported widely in the Western literature. Psychiatric diagnosis was also noted to identify types of mental illness reported by patients who were involved in suicidal behavior; therefore early diagnosis and prevention can be done. Knowing the methods used in suicide attempts is useful for future preventive work and finally the reasons for suicidal behavior will be useful for future research, especially in the area of risk and protective factors.

## 3. Results

A total of 38 studies from the year 1969 to 2011 fulfilled this review's inclusion criteria on attempted suicide in Malaysia (see Table 1). However, studies by Orr and Tin [7, 8] and Maniam [9, 10] used same subjects in both their papers. Therefore, the present review would treat each author's paper as one study, respectively. Sixty-three percent (63%) of the data were mainly gathered from government hospitals during their admission of suicide patients after the suicide attempt, and 34% were retrospective data from hospital charts and pathological records. The remaining 3% were gathered from subjects' hospital visits. A total of 10177 suicide attempters were studied across the 36 studies, sample sizes ranging from 4485 persons [11] to 1 person [12].

Six studies [13–18] used recognized scales to measure suicidal behaviors like the Suicide Intent Scale, Scale for Suicidal Ideation, Hopelessness Scale, and Reasons for Living Inventory. However, none of these studies validated these scales for use in a Malaysian context. Seven studies did not provide their sample classification according to gender [9–11, 15, 19–22], 3 studies only had female subjects [12, 16, 17], and one study [23] only had male subjects. Of the remaining studies, 24 studies [7, 8, 13, 14, 18, 24–43] reported more female (*N* = 2589) than male attempters (*N* = 992) and only one study [44] had more male attempters than female. According to the American Foundation for Suicide Prevention [45] women attempt suicide three times as often as men and this is consistent with findings on studies among suicide attempters in Malaysia. The fact that depression affects women about twice as often as men and that Malaysian women ranked third compared to men who ranked tenth on depression as the most disabling disease [3] might be a possible explanation for the higher rates of suicide attempts among females in Malaysia. While the age group of suicide attempters was not reported or unavailable in seventeen studies, the remaining studies reported that the highest number of attempters clustered within the age range of 20–30 years old. Sixteen studies (see Table 1, column 6) did not report the marital status of attempters. Study 31 was only conducted among married couples. Generally, there were more single attempters (*N* = 1031) than married (*N* = 701) ones. This may indicate that in Malaysia, marriage could perhaps serve as a protective factor from suicide behavior consistent with findings by Lorant et al. [46] and Nisbet [47]. On ethnicity, the highest numbers of suicide attempters were Indians (*N* = 1640), followed by the Chinese (*N* = 1208), Malays (*N* = 673), and other ethnicities. Maniam [48] listed some of the risk factors among Malaysian Indians which could explain the higher number of suicide attempts in this group including poverty (a majority of Indians are from the lower social class), alcoholism (this problem is the highest among the Indian ethnic group and it is well known as a contributory factor to the development of depression as well as ranking high as a risk factor for suicide), psychiatry morbidity, caste issues, other social distress, cultural and religious factors, and attitude to suicide. On the other hand, it is much more difficult for Muslims Malays to attempt suicide since it is against their religion. Education also contributed to some differences; 89% of the studies showed that suicide attempters had secondary level of education compared to primary and tertiary. However, this might merely reflect the fact that a majority of the population has had some secondary level of education. There were more employed suicide attempters (*N* = 656) than unemployed ones (*N* = 396). The remaining (*N* = 81) were students and homemakers. Unemployment has been associated with a higher likelihood of attempted suicide [49] while employment has been known to act as the protective factor for suicide attempters [50]. However, the protective effect of employment may not apply uniformly across the population, as studies indicate that there is a high level of job stress among workers in Malaysia [51].

One thousand and seven (17%) suicide attempters were diagnosed with some form of mental illness ranging from adjustment disorder to schizophrenia. Suicide methods used by the attempters include self-poisoning (89%) using weedkillers, pesticides, insecticides, household products, psychotropic drugs, and other chemicals. Suicide attempts using agricultural poisons were also high due to their easy availability, being often carelessly stored in high concentrations in farming communities with easy access to distressed people [27]. Other methods used by suicide attempters include wrist cutting, drowning, jumping from a height, and inflicting other self-inflicted injuries. Finally, reasons for attempting suicide were recorded in 11 studies and the most common reasons, 46% (*N* = 646), were due to conflicts with spouse, lover, and family members and at work place.

There were other interesting findings from this review. Nizam [13], for example, found that 74% of the suicide attempters in his study did not know how to access counseling services even when 53% of them have heard about such services from the media. Hussain and Zafri [31] also reported that 60% of married suicide attempters had been married for more than ten years. It was also noted that sixty percent of them had two or less children. Zuraida [32] focused on poor social network as a risk factor for suicidal behavior, emphasizing the importance of evaluating a patient's social support system as part of the management plan for suicide attempters. Meanwhile, Salleh et al.'s study [35] provided some evidence for the value of teaching patients coping skills in reducing future risk of suicide. This is consistent with Kannan et al.'s [18] findings showing how task-oriented coping skills, religious beliefs, and responsibility to family served as protective factors for patients in Kota Kinabalu, Sabah. Maisarah [17] also reported religiosity as the protective factor among suicide attempters. Other factors such as being a non-Malay, staying away from parents, media exposure, stress, poor coping skills, and not seeking professional help were found to be risk factors for suicide behavior among adolescents. Koh et al. [15] found that among suicide attempters in his study who were admitted to the University Malaya Medical Centre, the Indian population had the lowest scores on staying alive (not succumbing) after the attempt. The Indians were also found to have the highest scores on the Suicide Ideation Scale. Sorketti and Zuraida [37] reported that there were significant differences in the motives between those with self-poisoning and self-cutting. Meanwhile, Ainsah et al. [16] studied the relationship between the menstrual cycles and deliberate self-harm. The authors reported that deliberate self-harm was significantly associated with the menstrual cycle at the follicular phase, menarche with later onset, and menses with shorter duration. Personality traits of sensitivity, impulsivity, and worthlessness and personality disorders of paranoid and borderline types were found to be common in deliberate self-harm patients by Hamidin and Maniam [39]. The authors also reported that there was a high prevalence of life events among parasuicide patients when compared to medically ill patients, especially during the month prior to their admission to the hospital [43].

In summary, it should be noted that most (76%) of the studies on suicide attempt were descriptive studies that looked at sociodemographic data, psychiatric illness, and methods and reasons for suicide attempts. There is a clear need for more empirical studies that can explore suicide behavior in Malaysia in greater depth including exploring relationship between suicide attempt and mental illness, physical illnesses, risk factors, and protective factors. Another gap is the lack of scale validation that can give reliable and valid detection of suicidal behavior among Malaysians. Ideally, government needs to pay more attention to treating mental illnesses, especially depression, in the community in order to prevent suicidal behavior. Meanwhile, psychiatric and psychological services need to include behavioral management such as enhancing social support, problem solving skills, and coping skill techniques in their management. The decision by Malaysian government to ban paraquat in 2002 was one of the good movements to limit access to this product.

## 4. Discussion

It is evident from this review that research on suicide attempts in Malaysia is quite limited in areas like research design, statistical methodology, instrumentation, and intervention.

### 4.1. Limitations in the Study of Suicidal Behavior in Malaysia

(1) Malaysia is a multiracial society with Malays (54%), Chinese (25%), Indians (7.5%), and other ethnicities (13.5%). However, the impact of ethnicity on suicide attempt has not been studied systematically. Furthermore, Western scales have not been validated for use among non-Western people in Malaysia.

(2) In terms of sample size, many of the studies had inadequate sample sizes which might not report reliable results. The impact of gender has also been mostly overlooked.

(3) Another notable limitation is the lack of scale validation that can give reliable and valid detection of suicidal behavior among Malaysians. While Reasons for Living Inventory (RFL), Beck Suicidal Ideation Scale (BS1), and Beck Intent Scale (BIS) have been used in these studies in Malaysia, no attempt has been made to establish the reliability and validity of these scales for use in the Malaysian context. It is important that cultural and linguistic factors be taken into consideration, as these would affect the cut-off scores of some of the instruments for measuring suicidal behavior in Malaysia. Since these measures were derived from a Western perspective of understanding and investigation, they would seriously affect the interpretation of results in the Malaysian setting.

(4) A major gap in suicide research is in the area of treatment. There is no single study on treatment or effectiveness of risk management policies addressing suicide attempt. There are several reasons for this lack. One is that there is no specialised treatment/research facility for this difficult clinical group; the other could be due to lack of training/knowledge among the mental health professionals in conducting psychotherapy research, especially in a high risk population such as suicide attempters.

In summary, it is noted that most (76%) of the studies on suicide attempt were descriptive studies that looked at sociodemographic data, psychiatric illness, and methods and reasons for suicide attempts. There is a clear need for more empirical studies that can explore suicide behavior in Malaysia in greater depth including exploring relationship between suicide attempt and mental illness, physical illnesses, risk factors, and protective factors. Psychiatric and psychological services need to include behavioral management such as enhancing social support, problem solving skills, and coping skill techniques in their management. The decision by the Malaysian government to ban paraquat in 2002 seemed to be a good movement to limit access to this highly toxic product. However, in recent years, this ban has been withdrawn.

### 4.2. Future Directions in Research of Suicidal Behavior in Malaysia

Future research should focus on developing sound empirical research design and methodologies for studying suicidal attempt. This should include validating established instruments or measures for use in the Malaysian context. This process has started in Universiti Kebangsaan Malaysia. In addition, clinical and evaluation studies on treatment in the form of pharmacotherapy or psychological therapy ought to be included. There is also a need for researchers to focus on genetic/biological studies on suicidal behavior which is lacking in Malaysia. The impact of gender needs to be included in the studies. The impact of cultural differences on suicide behavior remains an interesting area of study in the Malaysian context and will benefit from a more systematic and empirical approach. Finally, there should be more effort to reach a wide range of research participants so that prevention of suicidal behavior can be planned at different levels.

## 5. Conclusion

If done more systematically, research on suicide attempts in Malaysia can shed light on the prevention and treatment of suicidal behavior in Malaysia. This needs to be addressed as a major public health concern. Suicide behavior contributes to a decrease in productivity and increase in national expenditure. Sound empirical research on suicidal behavior is an important element to suicide management.

## Figures and Tables

**Table 1:** Studies of attempted suicide in Malaysia.

Study	Methodology	Sample	Gender (*N*)	Age	Marital status	Ethnicity (%)	Education level	Employment	Psychiatric diagnosis	Method for suicidal behavior	Reasons
(1) Amarasingham and Lee (1969) [19]	Department of chemistry	666	NG	NG	NG	NG	NG	NG	NG	Arsenic (308) Formic acid (232) Caustic alkali (232) Organophosphorus insecticides (207) Chlorinated insecticides (115) Barbiturates (99) Sulphuric acid (61) Nonbarbiturate hypnotics (38) Ammonia (32) Acetyl salicylic acid (23) Methyl salicylate (21) Opium alkaloids (18) Hydrochloric acid (10)	NG

(2) Simons and Sarbadhikary (1972) [24]	Medical records	94	F (62) M (38)	NG	SL (58) MR (33) W/D/SP (10) PL (3)	CN (49) IN (22) ML (13) OT (10)	P (19) S (49) T (5) V (10) NK (11)	NG	Schizophrenia (37) Neurosis (30) Behavior disorder (15) Major effective illness (11) Mental retardation (1)	NG	NG

(3) Amarasingham and Hee (1976) [20]	Department of chemistry	620	NG	NG	NG	NG	NG	NG	NG	NG	NG

(4) Murugesan and Hock (1978) [44]	Hospital admission	94	F (24) M (70)	10–14 (5) 15–24 (62) 25–34 (18) 35–44 (7) 45–54 (1) 55> (1)	SL (54) MR (36) W/SP (3)	IN (62) CH (19) ML (11) OT (2)	P (39) S (40) T (2)	E (44) U (40)	Psychosis (6) Neurosis (20) Personality disorder (20) Alcohol addiction (6)	Insecticide (27) Psychotropics (23) Detergent (14) Iiniment (11) Others (20)	Domestic conflicts (31) Love affairs (29) Alcohol addiction (6) Illness (5) Work (4) Others (6)

(5) Haq and Buhrich (1980) [25]	Hospital admission	140	F (104) M (36)	15–20 (48) 22–31 (54)	SL (78) MR (54)	CH (59) IN (42) ML (39)	NG	NG	NG	Poisons (48) Drugs (73) Cutting, stabbing, hanging, and jumping (19)	Jilted (41) Marital problems (35) Family problems (23) Psychotic (13) Others (28)

(6) Yeoh (1981) [26]	Hospital admission	74	F (48) M (26)	10–15 (2) 16–25 (38) 26–35 (15) 36–45 (10) 46–55 (3) 55> (8)	SL (38) MR (31) SP/W (5)	CH (47) IN (20) ML (7)	NG	E (32) U (16) HW (19) ST (7)	Depressive neurosis (10) Other neuroses (1) Psychoses (12) Personality disorder (1) Organic psychosis (1) Drug addiction (5) Situational stress reaction (44)	Psychotropics (19) Insecticides (12) Liniment (5) Detergent (4) Analgesics (4) Narcotics (1) Knife wounds (11) Hanging (7) Jumping (6) Other drugs (2) Other objects (3)	Conflicts with elders (19) Health reasons (16) Marital conflict (12) Love disappointments (10) Financial problems, unemployment (2) Drug addiction (2) Others (10)

**(7) Orr and Tin (1985 a & b) [7, 8]	Hospital admission	271	F (211) M (60)	10–19 (54) 20–29 (149) 30–39 (42) 40–49 (15) 50–59 (7) 60–69 (4)	SL (135) MR (123) SP/D/W (13)	IN (149) CH (87) ML (35)	P (102) S (127) T (4) NN (30)	E (154) U (98) ST (17) PS (2)	NG	Tranquilizers and sedatives (84) Other oral medicines (34) Nonoral medicines (37) Harmful household compounds (54) Insecticides and weed killers (42) Physical injury (20) Unknown (19)	Marital problems (75) Family problems (64) Relationships problems (41) Medical illness (25) Psychiatric illness (23) Accidental (20) Other (23)

(8) Maniam (1988) [27]	Hospital records	134	F (81) M (53)	NG	NG	IN (182) CH (41) ML (6)	NG	NG	NG	NG	NG

(9) Ong amd Leng (1992) [21]	Hospital admission	273	NG	NA	NA	IN (130) CH (113) ML (21) OT (9)	NA	NA	NA	NA	NA

(10) Habil et al. (1992) [28]	Hospital admission	296	F (197) M (99)	0–19 (52) 20–39 (197) 40–59 (37) 60> (10)	SL (145) MR (134) SP/D/W (17)	IN (142) CH (120) ML (30) OT (4)	NG	NG	Adjustment disorder (176) Alcohol and drug dependence (38) Schizophrenia (30) Affective disorders and grief (25) Physical disorders and complaints (18) Personality disorders (8) Obsessive compulsive disorder (1)	Psychotropic (52) Analgesics (34) Other drugs (24) Detergent (35) Insecticide & pesticide (28) Agrochemicals (22) Other chemicals (7) Multiple drugs and chemical (49) Cutting and stabbing (18) Hanging (11) Ingestion of foreign objects like stones and blades (2) Burns (2) Combinations of physical and self-poisoning (12)	Depression form psychosocial stressors (175) Underlying schizophrenia (30)

(11) Azhar and Varma (1992) [23]	Hospital admission	3	M (3)	18 (1) 20 (1)	NG	NG	NG	NG	NG	NG	NG

**(12) Maniam (1994 a & b) [9, 10]	Hospital records	151	NG	NA	NA	IN (116) CH (29) ML (6)	NA	NA	NA	NA	NA

(13) Hussain and Hyman (1994) [29]	Hospital admission	195	F (142) M (53)	NA	NA	IN (93) CH (76) ML (23) OT (3)	NA	NA	NA	NA	NA

(14) Habil (1995) [30]	Hospital admission	99	F (72) M (17)	NA	NA	IN (54) CH (17) ML (13) OT (5)	NG	NA	NA	NA	NA

*(15) Nizam (1995) [13]	Hospital admission	78	F (59) M (19)	15–19 (23) 20–24 (23) 25–29 (12) 30–34 (10) 35–39 (4) 40–44 (4) 55–59 (1) 65–69 (1)	SL (55 ) MR (21) D/SP (1) W (1)	IN (46) CH (18) ML (12) OT (2)	T (2) S (72) P (1) NN (3)	NG	Psychoactive substance use disorder (1) Schizophrenia (1) Mood disorder (24) Impulse control disorder (1) Adjustment disorder with depressed mood (48)	Medicine (44) Chemicals (32) Injury (2)	Relief discomfort (16) Psychotic (1) Others (61)

(16) Maniam (1996) [12]	Hospital	1	F (1)	35	M (1)	NG	NG	E (1)	Depression (1)	Contracting AIDS	Depressed

(17) Hussain and Zafri (1997) [31]	Hospital admission	146	F (115) M (31)	21< (5) 22–30 (46) 31–40 (55) 41–50 (24) 51> (16)	M (146)	IN (68) CH (54) ML (23) OT (1)	NG	NG	Adjustment disorder (98) Major affective disorder (26) Alcohol-dependent syndrome (12) Schizophrenia (8) Premenstrual tension (2)	Overdose (74) Poisons ingestion (44) Physical harm (25) Multiple methods (3)	Extra marital affairs (42) Underlying psychiatry conditions (39) Problems due to other family members (22) Financial problems (16) Physical illnesses (11) Spouse abuse (16)

*(18) Peng and Chia (1997) [14]	Hospital admission	124	F (94) M (30)	11–15 (3) 16–20 (39) 21–25 (28) 26–30 (26) 31–35 (16) 36–40 (7) 41–45 (1) 46–50 (1) 51–55 (2) <55 (1)	MR (49) SL (64) D (8) C (3)	IN (64) CH (35) ML (21) OT (4)	P (30) S (85) T (8) NN (1)	E (82) U (33) ST (9)	Substance use disorder (5) Schizophrenia (7) Major depression (7) Affective disorder (2) Dysthymic disorder (23) Anxiety disorders (3) Adjustment disorders (3)	Poisoning (114) Injury (8) Both (2)	Claimed accidental (5) Relief of pain or discomfort (9) Intent self-harm (105) Psychotic (5)

(19) Zuraida (2000) [32]	Hospital admission	60	F (52) M (8)	NG	MR (26) SL (34)	IN (29) CH (19) ML (12)	P (1) S (43) T (6)	E (33) U/ST (13) HW (14)	Adjustment disorder with depressed mood (38) Acute stress reaction (11) Major depression (10) Schizophrenia (1)	NG	NG

(20) Siow (2001) [33]	Hospital records	16	F (11) M (5)	60–69 (10) 70–79 (6)	MR (6) SP (3) W (7)	CH (10) IN (5) OT (1)	P (6) S (4) NN (6)	NG	Major depression (13) Adjustment disorder with depressed mood (2) Chronic schizophrenia (1)	Drug overdose (5) Poison (5) Throat slashing (2) Cut wrists (2) Hanging (1) Jumping (1)	Quarrel with families (6) Ill health (5) Depressed (5)

*(21) Koh et al. (2002) [15]	Hospital admission	40	NG	NG	NG	ML (16) CH (14) IN (8) OT (2)	NG	NG	NG	Self-poisoning (92) Wrist-slashing and jumping from height (10)	NG

(22) Ab Rahman (2002) [22]	Medical records	51	NG	NG	NG	NG	NG	NG	NG	NG	NG

(23) Kok et al. (2003) [34]	Hospital admission	330	F (219) M (111)	15< (26) 16–20 (105) 21–30 (110) 31–40 (57) 41–50 (19) 51> (13)	NG	CH (139) ML (109) IB (36) BD (26) OT (20)	NG	NG	Depressive features (47) Schizophrenia (11)	NG	NG

(24) Salleh et al. (2005) [35]	Hospital admission	50	F (40) M (10)	16–25 (30) <30 (34)	SL (27) MR (17) D (5) W (5)	IN (40) CH (16) ML (12) OT (2)	NG	E (20) U (30)	Dysthymia (4) Major depression (15) Adjustment disorder (30)	NG	NG

(25) Fathel et al. (2005) [36]	Hospital record	249	F (177) M (72)	NG	NG	CH (118) ML (43) IN (78)	NG	NG	NG	NG	NG

(26) Sorketti and Zuraida (2007) [37]	Hospital admission	77	F (57) M (20)	NG	MR (49) SL (20)	CH (37) IN (29) ML (11)	NG	E (36) U (36)	Adjustment disorder (38) Major depression (24) Other diagnoses (15)	Self -poisoners (52) Self-cutters (25)	Relationship problems (54) Employment problems (12) Health-related problems (11)

(27) Rajasuriar et al. (2007) [11]	Hospital records	448 5	NG	NG	NG	NG	NG	NG	NG	NG	NG

(28) Fathelrahman et al. (2008) [38]	Hospital admission	320	F (225) M (95)	>45 (295)	NG	NG	NG	NG	NG	NG	NG

*(29) Ainsah et al. (2008) [16]	Hospital admission	86	F (86)	18–23 (50) 24–29 (23) 30–35 (3) 36–44 (7) 45 (3)	MR (34) SL (52)	ML (36) IN (32) OT (18)	NN/P (19) S (46) T (21)	E (67) U (19)	Major depression (20) Dysthymia (7) Panic disorder (3) Agoraphobia (1) Comorbid diagnoses (4) Alcohol abuser (1) Substance abuser (1) Bulimia nervosa (2) Generalized anxiety disorder (1)	Self-poisoning (78) Self-injury (6) Both (2)	NG

(30) Hamidin and Maniam (2008) [39]	Hospital admission	50	F (39) M (11)	NG	MR (15) SL (35)	IN (26) ML (20) CH (4)	S (33)	NG	Major depressive disorder (11) Dysthymia (1) Alcohol abuse (2) Generalized anxiety disorder (1) Comorbidity (5)	Self-poisoning (48) Self-injury (2)	NG

(31) Teo et al. (2008) [40]	Hospital records	189	F (137) M (52)	<20 (68) 21–30 (69) 31–40 (35) 41–50 (9) 51–60 (5) >60 (3)	NG	IN (122) CH (35) ML (25) FR (7)	NG	NG	NG	Drugs (83) Household products (22) Pesticide (55) Carbon monoxides (4) Drowning (4) Hanging (23) Injuries by sharp objects (2) Others (39)	Problems Relationship (108) Health (18) Financial (6) At work (2) At school/exams (1) Not recorded (54)

*(32) Maisarah (2008) [17]	Hospital admission	80	F (80)	≤20 (25) >20 (55)	SL (64) MR (16)	ML (36) IN (35) CH (9)	P (6) S (47) V (7) T (20)	E (64) U (16)	NG	Drugs overdose (48) Insecticide & pesticide (16) Household products (13) Wrist cutting (3)	NG

*(33) Kannan et al. (2010) [18]	Hospital admission	42	F (39) M (3)	NG	SL (24) MR (13) D (3) SP (1)	KD (12) ML (4) CH (3) BJ (15) SB (5) IND (3)	NG	E (33) U (9)	NG	NG	NG

(34) Zyoud et al. (2010) [41]	Hospital records	177	F (149) M (28)	<20 (66) 20–30 (88) >30 (23)	SL (140) MR (34) D (3)	ML (89) IN (43) CH (40) OT (5)	NG	E (91) U (86)	NG	NG	NG

(35) Zyoud et al. (2010) [42]	Hospital records	280	F (235) M (45)	NG	NG	NG	NG	NG	Major depression (30) Adjustment disorder (128) Anxiety disorder (10)	NG	NG

(36) Hamidin and Maniam (2011) [43]	Hospital admission	50	F (39) M (11)	NG	MR (15) SL (35)	IN (26) ML (20) CH (4)	S (33)	NG	Major depressive disorder (11) Dysthymia (1) Alcohol abuse (2) Generalized anxiety disorder (1) Comorbidity (5)	Self-poisoning (48) Self-injury (2)	Personal illness issues (1) Family illness (11) Interpersonal issues (47) Work issues (12) Financial (14) Others (8)

*Used recognized psychological instrument to measure suicide attempt.

**Used same subjects in two published articles. Therefore, reported as a single study in this paper but listed as 2 different studies in numbering of references.

Notes: *N*: number; SP: separated; CN: Chinese; KD: Kadazan; U: unemployed; P: primary; NA: not available; PL: polygamy; IN: Indian; BJ: Bajau; HW: housewife; S: secondary; NN: none; SL: single; ML: Malay; IB: Iban; ST: students; T: tertiary; NG: not given; MR: married; SB: Sabahan; BD: Bidayuh; OT: others; V: vocational; NK: not known; W: widow; IND: Indonesian; F: female; E: employed; IE: informal education; C: cohabit; FR: foreigner; M: male; D: divorce.
